# Lung inhomogeneities, inflation and [^18^F]FDG uptake rate in ards

**DOI:** 10.1186/2197-425X-3-S1-A90

**Published:** 2015-10-01

**Authors:** M Cressoni, D Chiumello, M Brioni, I Algieri, M Gotti, K Nikolla, D Massari, A Cammaroto, A Colombo, P Cadringher, E Carlesso, L Gattinoni

**Affiliations:** Dipartimento di Fisiopatologia Medico-Chirurgica e dei Trapianti, Fondazione IRCCS Ca’ Granda-Ospedale Maggiore Policlinico, Università degli Studi di Milano, Milan, Italy; Dipartimento di Anestesia, Rianimazione ed Emergenza Urgenza, Fondazione IRCCS Ca' Granda-Ospedale Maggiore Policlinico, Milan, Italy

## Introduction

In ARDS lung parenchyma presents great variability in inflation, lung inhomogeneities and [^18^F]FDG uptake. In fact, inflation progressively decreases along the sternum-vertebral axis[^1^] leading to further inhomogeneities that may act as “stress raiser”.[^2^] That can activate a local inflammatory response leading to edema.

## Objectives

We aimed to examine the voxel by voxel relationship between [^18^F]FDG uptake and inhomogeneity according to the actual classification of ARDS.

## Methods

20 ARDS patients underwent a PET-CT scan at 10 cmH_2_O. [^18^F]FDG uptake was determined with the graphical Patlak approach[3] voxel by voxel. Lung inhomogeneities were determined by measuring the gas/tissue ratio in two contiguous lung regions. We defined inhomogeneities the fraction of lung volume whose inhomogeneities were greater than 1.61.[4]

## Results

5 patients presented mild, 12 moderate and 3 severe ARDS. In mild and moderate ARDS a consistent lung fraction is homogeneous with a high [^18^F]FDG metabolic activity (53 ± 14% and 53 ± 20%). Inhomogeneous lung fraction with a higher [^18^F]FDG uptake increases from mild to severe (12 ± 3%, 16 ± 9% and 27 ± 11%). On the other hand, the homogeneous parenchyma with normal [^18^F]FDG uptake decreases in worse ARDS (33 ± 14%, 26 ± 20% and 5 ± 9%).

## Conclusions

Our findings indicate that the actual classification of ARDS from mild to severe reflects the underlying pathophysiology. In fact, while a similar sized homogeneous and inflamed/metabolically more active compartment is present in all the ARDS patients, in mild ARDS it is associated with a consistent fraction of normal lung while in severe ARDS is primarily associated with inhomogeneous, inflamed/metabolically more active lung tissue.
